# Lowering the Risk of Rectal Cancer among Habitual Beer Drinkers by Dietary Means

**DOI:** 10.4061/2011/874048

**Published:** 2011-06-08

**Authors:** Gabriel Kune, Lyndsey Watson

**Affiliations:** ^1^Faculty of Medicine, Dentistry and Health Sciences, University of Melbourne, 41 Power Street, Toorak, VIC 3142, Australia; ^2^Mother and Child Health Research, La Trobe University, Melbourne, VIC 3000, Australia

## Abstract

Whole-life beer consumption and a quantitative measurement of several dietary micronutrients consumed in adult life were obtained from the dietary and alcohol data of the case-control arm of the population-based *Melbourne Colorectal Cancer Study*. There was a statistically significant risk, adjusted for other established risk factors, among habitual beer drinkers (AOR 1.75, 95% CI 1.28–2.41) with a significant positive dose-response effect (AOR trend 1.34, 95% CI 1.16–1.55). Among beer consumers the data were interpreted as showing an attenuation of this risk with consumption of the four micronutrients involved in methylation: folate, methionine, vitamins B6 and B12, and the four micronutrients examined with antioxidant properties: selenium, vitamins E, C, and lycopene. The strongest effects were noted with vitamins E, C, and lycopene, and the weakest with methionine and selenium. Whilst not condoning excessive beer drinking, the regular consumption of foods rich in these micronutrients may provide a simple and harmless preventative strategy among persistent habitual beer drinkers and deserves further study with larger study numbers.

## 1. Introduction

Regular beer drinking is a risk factor for cancer of the rectum [1–5]. Kune and colleagues were the first to report in 1987 that this risk was annulled by a high consumption of vitamin-C-containing foods [6], and subsequently several studies indicated that the alcohol risk was significantly attenuated by a high folate and possibly high methionine intake [7–10]. The objective of this study was to assess whether the dietary micronutrients involved in one-carbon metabolism, namely, folate, methionine, vitamins B6 and B12, and micronutrients with antioxidant properties particularly vitamin C, vitamin E, lycopene, and selenium, might counteract the elevated risk of rectal cancer among habitual beer drinkers.

## 2. Materials and Methods

The data were obtained from the case-control arm of the population-based *Melbourne Colorectal Cancer Study *[11]. The principal alcohol findings were a significant risk for rectal cancer among habitual beer drinkers, a risk which was annulled by a high consumption of vitamin-C-containing foods [6]. The main dietary findings were published in 1987 [12]. The micronutrient analysis of colorectal cancer risk we reported in 2006 [13], but this did not include the risks for rectal cancer associated with micronutrient intake among beer drinkers, therefore the data here represent unpublished material. 

The study included 323 incident cases of rectal cancer and 727 age/sex frequency matched randomly chosen community controls in Melbourne, Australia, collected in 1980-1981. There was a quantitative estimate of all foods eaten during a period most representative of the previous 20 years [12]. Whole-life beer consumption was estimated and categorised into tertiles of drinking based on all the study participants consuming beer. Alcohol data were missing for 7 cases and 3 controls, and these were excluded from the analysis. Rectal cancer risk associated with each level of beer consumption was estimated (Tables 1 and 2).

The intake of micronutrients involved in one-carbon metabolism (folate, methionine, and vitamins B6 and B12), and four other micronutrients with antioxidant properties (vitamins C, E, lycopene, and selenium) were estimated from foods eaten using the US Department of Agriculture nutrient tables [14], and categorised into quintiles of consumption based on all the participants in the study. Nutritional supplements were not included. Logistic regression was used to estimate associations as odds ratios (ORs) between rectal cancer and the quintiles of micronutrient intake in beer drinkers taken as a single category, to demonstrate *explicitly* the effects investigated. Adjusted ORs (AORs) were obtained by adding the variables age, sex, alcohol (yes/no), body mass index (quintiles), energy intake (quintiles), family history of colorectal cancer (yes/no), oral contraceptive pill use (yes/no), cigarette pack years (none, 1–19, 20–39, ≥40), aspirin use yes/no), and NSAID use (yes/no) to the micronutrient logistic regression model, and level of beer consumption also added to the model of Table 3.

To investigate the hypothesis that micronutrients may attenuate the risk of rectal cancer in beer drinkers, the data were stratified by beer consumption, and subgroup analyses were undertaken of beer consumers and nonconsumers.

## 3. Results

Range of beer consumption per day in tertiles, and the number and percentage distribution by rectal cancer cases and controls is shown in Table 1. Habitual beer drinking was again confirmed to be a statistically significant risk for rectal cancer, and remained so after adjustments were made for the several risk factors found in the study, as shown in Table 2 (AOR 1.75, 95% Confidence Interval (95% CI) 1.28, 2.41, *P* = .001). There was also a statistically significant dose-response effect with increasing levels of beer consumption as seen in Table 2 (AOR trend 1.34, 95% CI 1.16–1.55, *P* ≤ .001). 

Among beer consumers the dietary micronutrients involved with methylation (folate, methionine, and vitamins B6 and B12), with one exception, had AORs around or below 1. The exception was the elevated AOR in the two highest quintiles of methionine consumption (Table 3). All were null results, none statistically significant at the  .05 level. Similar results were noted among non-consumers of beer, with the exception of the third and fourth quintiles of folate, and fourth quintile of vitamin B6 where protective effects were present which were statistically significant (Table 3). 

Turning to the four micronutrients with antioxidant properties, selenium, vitamin E, vitamin C, and lycopene among beer consumers, with one exception (fifth quintile of selenium), all had AORs around or below 1 (Table 3). Statistically significant protective effects were noted in all categories of vitamin E consumption and among the highest quintile of vitamin C and the fourth and fifth quintiles of lycopene consumers (Table 3). Among non-consumers of beer the AORs were around or below 1, with statistically significant protective effects in the third and fourth quintile of selenium, third quintile of vitamin E, and virtually all levels of vitamin C consumption (Table 3).

## 4. Discussion

Quantitative data of rectal cancer risk adjusted for known risk factors in habitual beer drinkers linked to various micronutrients are sparse, making comparisons with our data difficult. As already mentioned, several studies found that the alcohol risk was significantly attenuated by a high consumption of folate and methionine [7–10]. One other study investigated the alcohol risk (but not beer risk specifically) in colorectal cancer in relation to the four known micronutrients involved in methylation processes, namely, folate, methionine, and vitamins B6 and B1, and found a null result [15], however there were few alcohol consumers and few rectal cancers in the cohort. 

Conclusions that can be drawn from our data are that vitamin E, the highest quintile of vitamin C, and the two highest quintiles of lycopene have significant protective effects against the rectal cancer risk among beer consumers. The data are also consistent with beer drinking and micronutrient consumption having opposing but independent effects on rectal cancer risk. The four micronutrients involved in methylation processes (folate, methionine, and vitamins B6 and B12), have AORs around or below 1 (with the exception of the two highest quintiles of methionine which are a risk) uniformly indicating an attenuation of the rectal cancer beer risk by these micronutrients. We believe that the lack of statistical significance is due to the relatively small number of beer consumers with rectal cancer. Additionally, the previously reported major diet and alcohol findings of this Melbourne study have largely been in accord with other studies [1–6, 12, 13]. Methionine is mainly found in high protein foods, including red meat, which has previously been found in our study to be a statistically significant risk for rectal cancer [12]. These data lend some indirect support to the available evidence that beer is a risk for rectal cancer, at least in part because together with other alcoholic drinks it is regarded as a methyl antagonist interfering with DNA synthesis and repair, thereby promoting colorectal tumour formation [2, 16, 17].

Similarly, the four micronutrients with antioxidant properties, selenium, vitamins E, C, and lycopene, are consistent with the counteraction of the increased risk of rectal cancer in beer drinkers, with null results for selenium and the lower quintiles of vitamin C and lycopene, and additionally a statistically significant protective effect for all quintiles of vitamin E, the highest quintile of vitamin C, and the two highest quintiles of lycopene containing foods (Table 3). Whilst so-called “oxidative stress” or “oxidative damage” to DNA caused by several exposures including alcohol has been invoked as a possible agent in carcinogenesis theory [18–20], there is no direct evidence to our knowledge of this mechanism in rectal cancer. However, oxidative damage by alcohol and counteraction by micronutrients with antioxidant properties is a possible explanation for the interpretation of our data.

## 5. Conclusions

Habitual longstanding beer drinking was associated with a statistically significant risk for rectal cancer, and there was a significant positive dose-response effect. As measured in this study, the regular consumption of foods containing the micronutrients involved with methylation, folate, methionine, vitamins B6 and B12, and the four micronutrients examined with antioxidant properties, selenium, vitamins E, C, and lycopene, all attenuated this risk among beer drinkers. The strongest effects were noted for vitamins E, C and lycopene, and the weakest for methionine and selenium containing foods. 

Whilst certainly not encouraging excessive beer drinking, the results support the proposition that the regular consumption of foods containing some of the micronutrients involved in one-carbon metabolism, particularly folate, vitamins B6, B12, and some with antioxidant properties, namely, vitamins E, C, lycopene and selenium, micronutrients that are plentiful in many vegetables, fruits, grains, nuts, and fish, may provide a relatively simple and harmless means of counteracting the excess risk of rectal cancer in habitual and persistent beer drinkers. This conclusion in our view deserves further investigation with larger study numbers and therefore greater statistical power.

## Figures and Tables

**Table 1:** Upper levels of daily consumption of beer, number, and percentage distribution by controls and rectal cancer cases in the Melbourne Colorectal Cancer Study.

	Rectal cancer cases		Controls	
Beer				
Upper levelof intake/dayof category (ml/day)	N 316	%	N 724	%
0	175	*55*	490	*68*
1–200	40	*13*	85	*12*
201–740	50	*16*	80	*11*
More than 740	51	*16*	69	*10*

**Table 2:** Odds ratios (ORs), 95% confidence intervals (95% CIs) and *P* values for categories of beer consumption for rectal cancer unadjusted and adjusted for other risk factors. Data from the Melbourne Colorectal Cancer Study.

Upper levelof intake/dayof category (ml/day)	Unadjusted^(a)^		Adjusted^(b)^	
	OR^(c)^	(95% CI)	OR^(c)^	(95% CI)
Beer intake				
None	1.00		1.00	
Any	1.68***	(1.25,2.27)	1.75***	(1.28,2.41)
1–200 ml/day	1.29	(0.84,1.98)	1.34	(0.86,2.07)
201–740 ml/day	1.83**	(1.21,2.79)	1.99**	(1.28,3.08)
More than 740 ml/day	2.15***	(1.38,3.36)	2.28***	(1.40,3.69)
Trend	1.31***	(1.14,1.50)	1.34***	(1.16,1.55)

(a) Adjusted for age and sex only.

(b) Adjusted for age and sex, alcohol (yes/no), body mass index (quintiles), energy intake (quintiles), family history of colorectal cancer (yes/no), oral contraceptive pill use (yes/no), and cigarette pack-years (none, 1–19, 20–39, or ≥40), aspirin (yes/no), and NSAIDS (yes/no).

(c) **P ≤ .01, ***P ≤ .001.

**Table 3:** Association between quintiles of micronutrient intake and rectal cancer amongst beer consumers and nonconsumers.

			Beer consumers			Non-consumers of beer		
Micronutrient			AOR^a^	(95% CI)	*P*	AOR^a^	(95% CI)	*P*
Folate (mcg/day)	1	246	1.00			1.00		
	2	297	0.47	(0.15,1.43)	.2	0.68	(0.42,1.13)	.14
	3	347	0.51	(0.17,1.48)	.2	0.48	(0.28,0.85)	.01
	4	419	0.51	(0.18,1.45)	.2	0.56	(0.30,1.03)	.06
	5	1367^b^	0.87	(0.27,2.78)	.8	0.89	(0.44,1.79)	.7
Methionine (g/day)	1	1.3	1.00			1.00		
	2	1.6	1.06	(0.43,2.57)	.9	0.69	(0.41,1.17)	.2
	3	1.9	0.79	(0.32,1.96)	.6	0.70	(0.38,1.29)	.2
	4	2.3	1.73	(0.65,4.59)	.3	0.72	(0.35,1.47)	.4
	5	6.0	2.42	(0.85,6.88)	.10	1.21	(0.52,2.82)	.7
Vitamin B6 (mcg/day)	1	1.7	1.00			1.00		
	2	2.1	0.41	(0.13,1.31)	.13	0.66	(0.39,1.12)	.13
	3	2.6	0.51	(0.16,1.58)	.2	0.56	(0.30,1.03)	.06
	4	3.4	0.62	(0.19,2.06)	.4	0.42	(0.22,0.79)	.007
	5	12.7	0.49	(0.14,1.64)	.2	0.57	(0.29,1.12)	.10
Vitamin B12 (mg/day)	1	4.1	1.00			1.00		
	2	5.7	1.07	(0.43,2.63)	.9	1.60	(0.95,2.69)	.08
	3	7.6	0.66	(0.27,1.60)	.4	0.89	(0.49,1.60)	.7
	4	11.1	0.74	(0.30,1.81)	.5	0.68	(0.38,1.22)	.2
	5	39	0.47	(0.19,1.15)	.10	0.64	(0.34,1.20)	.2
Selenium (mcg/day)	1	80	1.00			1.00		
	2	99	0.58	(0.18,1.83)	.3	0.70	(0.41,1.17)	.2
	3	118	0.92	(0.32,2.60)	.9	0.49	(0.27,0.90)	.02
	4	145	0.66	(0.21,2.04)	.5	0.47	(0.24,0.93)	.03
	5	379^b^	1.21	(0.37,3.90)	.8	0.61	(0.28,1.29)	.2
Vitamin E (mg/day)	1	5.1	1.00			1.00		
	2	7.1	0.35	(0.16,0.78)	.01	0.66	(0.37,1.17)	.2
	3	9.3	0.40	(0.18,0.88)	.02	0.42	(0.23,0.77)	.005
	4	12.7	0.44	(0.21,0.93)	.03	0.69	(0.39,1.24)	.2
	5	36	0.35	(0.15,0.80)	.01	0.65	(0.36,1.14)	.13
Vitamin C (mg/day)	1	69	1.00			1.00		
	2	104	0.85	(0.42,1.75)	.7	0.52	(0.29,0.94)	.03
	3	141	0.92	(0.45,1.88)	.8	0.58	(0.32,1.04)	.07
	4	198	0.53	(0.25,1.10)	.09	0.55	(0.31,0.98)	.04
	5	1036^b^	0.26	(0.11,0.60)	.002	0.48	(0.26,0.87)	.02
Lycopene (mcg/day)	1	280	1.00			1.00		
	2	706	0.55	(0.25,1.22)	.14	1.04	(0.60,1.78)	.9
	3	1336	0.77	(0.36,1.61)	.5	1.15	(0.66,2.00)	.6
	4	2731	0.47	(0.23,0.98)	.05	1.13	(0.64,2.00)	.7
	5	22973^b^	0.38	(0.18,0.81)	.01	1.25	(0.70,2.23)	.4

^a^Adjusted for age and sex, alcohol (yes/no), body mass index (quintiles), energy intake (quintiles), family history of colorectal cancer (yes/no), oral contraceptive pill use (yes/no), cigarette pack, years (none, 1–19, 20–39, or ≥40), aspirin (yes/no), NSAIDS (yes/no), and beer consumption (1–200, 201–740, or >740 ml/day).

^b^90 percentile for intake per day for folate is 500 mcg, for selenium is 175 mcg, for vitamin C is 250 mg and for lycopene is 4600 mcg.
